# CD99/MIC2 Constitutes a Differentiation Antigen of a Human Osteoblast Cell Line

**DOI:** 10.4021/wjon415w

**Published:** 2011-12-19

**Authors:** Gerhard Hamilton, Ulrike Olszewski-Hamilton

**Affiliations:** a Ludwig Boltzmann Cluster of Translational Oncology, Nussdorferstrasse 64/6, A-1090 Vienna, Austria

**Keywords:** Osteoblast, Differentiation, CD99, MIC2, HBA-71, Ewing’s sarcoma, Histone deacetylase inhibitor

## Abstract

**Background:**

The histological origin of the Ewing’s family of tumors (EFT) is still not clear. Since these small cell bone tumors may originate from osteogenic stem cells, the presence of the CD99/MIC2 antigen, known to be overexpressed in EFT, was studied in a human osteoblast cell line in response to differentiation inducers.

**Methods:**

The HBA-71 monoclonal antibody directed to the CD99/MIC2 antigen was used to stain a human osteoblast cell line as well as the two EFT cell lines KAL and EW-2 after pretreatment of the cells with the differentiation inducers calcitriol and the histone deacetylase (HDAC) inhibitors sodium butyrate (NaB), sodium phenylacetate (NaPA) as well as N, N’-hexamethylen-bis-acetamide (HMBA). Alkaline phosphatase (ALP) levels were determined as cellular differentiation marker.

**Results:**

Significant expression of the CD99/MIC2 antigen, yielding a molecular weight of 32 kD in Western blotting, was found in the human osteoblast cell line. Pretreatment of the osteoblasts with calcitriol and HMBA increased ALP content and suppressed the CD99/MIC2 antigen. Calcitriol had no major effect on CD99/MIC2 expression of both EFT cell lines, but HMBA enhanced ALP activity in KAL cells and downregulated CD99/MIC2. EW-2 cells exhibited reduced levels of both CD99/MIC2 and ALP.

**Conclusions:**

This study supports the role of CD99/MIC2 as differentiation antigen of osteoblasts and a Ewing’s sarcoma cell line with neuroectodermal phenotype. Response to calcitriol is absent or low in the two EFT cell lines tested.

## Introduction

Ewing’s sarcoma (ES) and peripheral/primitive neuroectodermal tumor (PNET), comprising the Ewing’s family of tumors (EFT), are related rare cancers of childhood and adolescence characterized by early dissemination and poor prognosis despite aggressive surgical and chemotherapeutic treatment [1]. In 1921, James Ewing first described this malignant round cell bone tumor and since then various cells, such as mesenchymal, myeloid, reticulum, pericytic, neuroepithelial and primitive multipotential cells have been suspected as the cells of origin [2]. In search of antigens selective for ES/PNET versus neuroblastoma the monoclonal HBA-71 antibody was established and found to recognize a strongly expressed cell surface protein of ES/PNET, besides normal tissues like cortical thymocytes, islets of Langerhans, granulosa and Sertoli cells [3]. Prompted by the development of the HBA-71 antibody the corresponding antigen was identified as MIC2 gene product by two independent groups [4, 5]. MIC2 is the product of a pseudoautosomal gene in humans which encodes an 18 kD transmembrane protein and later on was clustered as CD99 antigen [6]. In it’s heavily glycosylated form (30/32 kD) CD99/MIC2 is expressed in small amounts on almost every human cell type. Identification of CD99/MIC2 as an EFT-associated marker has greatly facilitated the differential diagnosis of these cancers, besides specific chromosomal translocations coding for Ewing sarcoma gene (EWS) fusion proteins [1]. The EFT with typical EWS gene rearrangements exhibit CD99/MIC2 expression; however, the histogenetic origin of EFT is not clear. The rate-limiting EWS rearrangement by random fusion with Fli1 or other Ets transcription factor genes is likely to occur in a bone-associated CD99/MIC2-positive normal cell type. Since detection of antigens in processed bone tissue is hard to achieve and subpopulations are difficult to discriminate in the presence of CD99/MIC2-positive bone marrow cells and unspecific background staining, we checked an immortalized human osteoblast line grown in vitro for HBA-71/CD99 reactivity. The AHTO-7 (adult human trabecular osteoblast-7) cell line was selected from several clones obtained by immortalization of adult normal human trabecular bone cells using the SV 40 large T antigen and expresses alkaline phosphatase (ALP), osteocalcin and collagen I as characteristic markers of the differentiated phenotype of the original tissue [7].

## Methods

### Cell lines and culture conditions

Human AHTO-7 cells (passage number 21) were provided by Dr. A. Lomri (INSERM Unit 349, Cellular and Molecular Biology of Bone and Cartilage, Paris, France). This osteoblast cell line, as well as the KAL and EW-2 EFT cell lines and HBA-71/isotype control NIC-1 murine hybridoma cells were cultured in 10% fetal bovine serum/RPMI-1640 medium (Seromed, Berlin, Germany) supplemented with 4 mM glutamine. The osteoblast cell line was harvested using calcium/magnesium-free phosphate-buffered saline (PBS, Gibco Invitrogen, Paisley, Scotland; approximately 20 minutes, room temperature).

### Chemicals

Except indicated otherwise, all chemicals were purchased from Sigma-Aldrich, St. Louis, MO, USA. The following differentiation inducers were used: 1α, 25-Dihydroxyvitamin D_3_ (1, 25-vitD3, Calcitriol), sodium phenyl acetate (NaPA, Calbiochem, La Jolla, CA), sodium butyrate (NaB) and N, N’-hexamethylen-bis-acetamide (HMBA).

### Immunofluorescence tests

1 x 10^5^ cells in 100 µL medium/well were labeled using an equal volume of HBA-71 IgG1 tissue culture supernantant at 4 °C for 30 minute. Cells were washed and incubated with anti-mouse IgG (Fab-specific)-FITC as secondary reagent. Fluorescence was analyzed using an Epics XL flow cytometer (Coulter, Miami, FL, USA).

### Cell proliferation assays

Cells were harvested, counted (Coulter counter, Miami, FL, USA) and transferred to the wells of microtiter plates (96 wells, Costar, Cambridge, MA, USA) in a cell density of 10^4^ cells/well in 100 µL medium. Appropriate dilutions of test compunds were added to a total volume of 200 µL/well and the plates incubated for four days under tissue culture conditions. Cell proliferation was quantitated using a modified tetrazolium salt assay (EZ4U, Biomedica, Austria) and measurement of the reduced dye at 450 nm (ELISA reader, Eurogenetics, Brussels, Belgium).

### Determination of ALP activity

Cells (5 x 10^4^/well, 2 ml medium) were treated with 1, 25-vitD3 or histone deacetylase inhibitor (HDAC) differentiation inducers in 24-well plates (Costar, Cambridge, MA, USA) for three days. Medium was removed, the cells washed with PBS and cell layers were covered with 300 µL 0.1% Triton X 100/phosphate buffer (pH = 10.5) and stored frozen at - 80 °C. When samples were thawed, the extracts were scraped and distributed to three wells of 96-well microtiter plates and p-nitrophenylphosphate substrate was added. The amount of p-nitrophenol released as indicator of ALP activity was measured in a microplate reader at 415 nm. Cell numbers were counted in control cultures incubated under identical conditions.

### Western blotting of CD99/MIC2 antigen using HBA-71 antibody

Cells were lysed using a buffer consisting of 0.14 M NaCl, 0.2 M triethanolamine, 0.5% NP40, 0.2% deoxycholate, 1 mM phenylmethylsulfonyl fluoride, 4 µg/mL leupeptin, 4 µg/mL aprotinin and centrifuged. Following protein determination (Micro BCA assay kit; Pierce, Rockford, IL, USA) extracts were separated on 8% sodium dodecylsulfate gels and blotted to nitrocellulose membranes (Trans-Blot; Biorad, Hercules, CA, USA). HBA-71 supernatant was diluted 1:10 in 0.5% bovine serum albumin/PBS and used as primary antibody. Anti-β-actin monoclonal antibody was used for control. After washing secondary antibody (anti-mouse-HRP-conjugate, 1:10 000; Promega, Madison, WI, USA) was applied followed by SuperSignal substrate (Pierce) and exposition on HyperFilm MP, (GE Healthcare, Little Chalfont, UK).

### Statistical analysis

Data were analysed for statistical differences by t-tests and p values < 0.05 were regarded as significant. Calculations were done using Origin 7.5 software (Origin, Northampton, MA, USA).

## Results

### HBA-71 immunofluorescence reactivity of AHTO-7 cells

**Figure 1:**
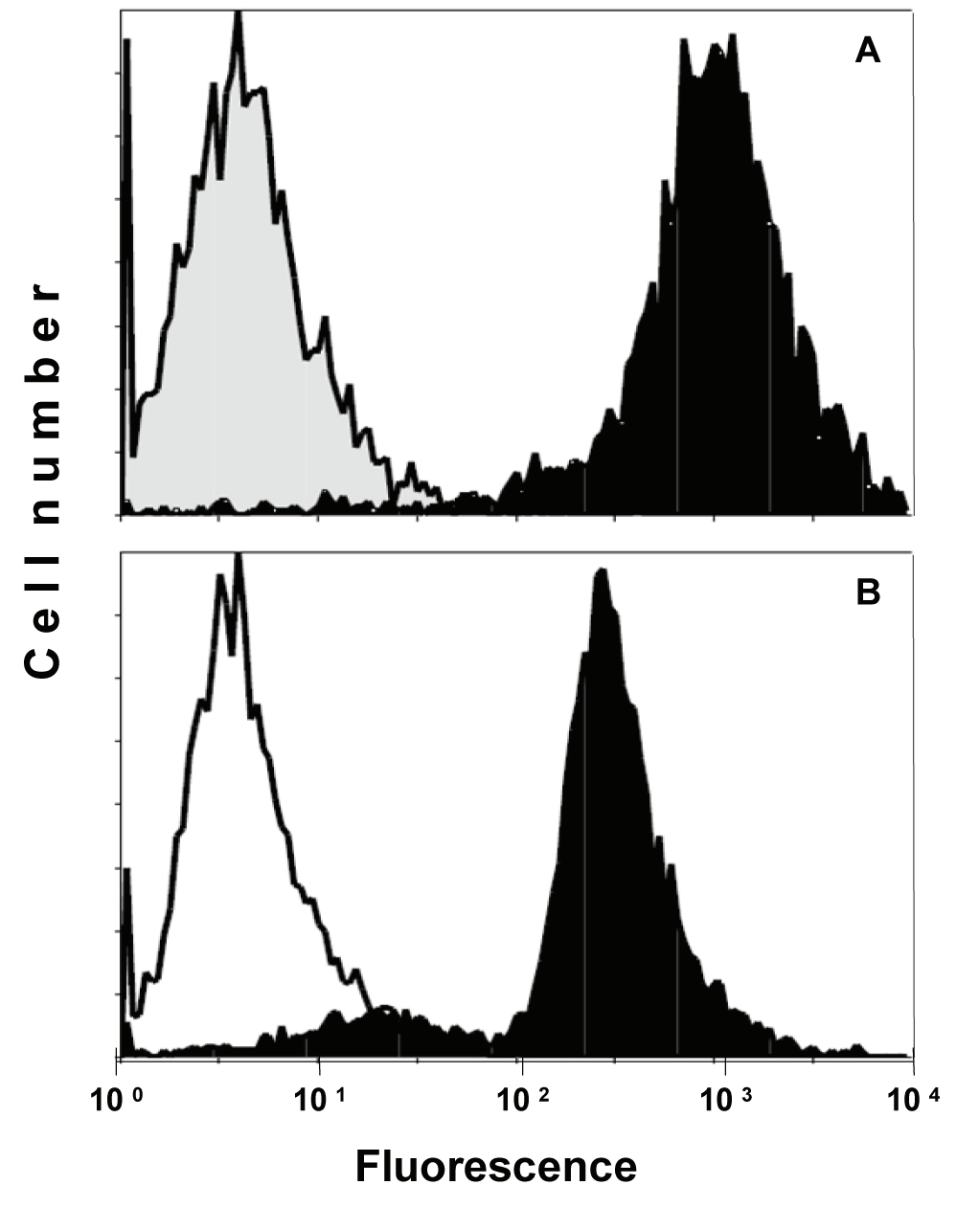
Flow cytometric analysis of CD99/MIC2 HBA-71 antigen expression of (A) KAL EFT cells and (B) AHTO-7 osteoblasts. Immunofluorescence of the cells stained with HBA-71 is shown in black, the NIC-1 isotype controls in grey (KAL) and white (AHTO-7), respectively.

Expression of CD99/MIC2 of AHTO-7 osteoblasts was detected using the mouse monoclonal antibody HBA-71, which had been developed in our lab. Figure 1 shows the flow cytometric analysis of the CD99/MIC2-positive EFT cell line KAL as control (A) and AHTO-7 cells (B) stained with this antibody in indirect immunofluorescence. The osteoblasts exhibited a high expression of CD99/MIC2. Fluorescence of KAL cells was five times stronger under the same conditions; however, these tumor cells are much larger than the AHTO-7 cells.

### Modulation of HBA-71 reactivity of AHTO-7 cells

**Figure 2:**
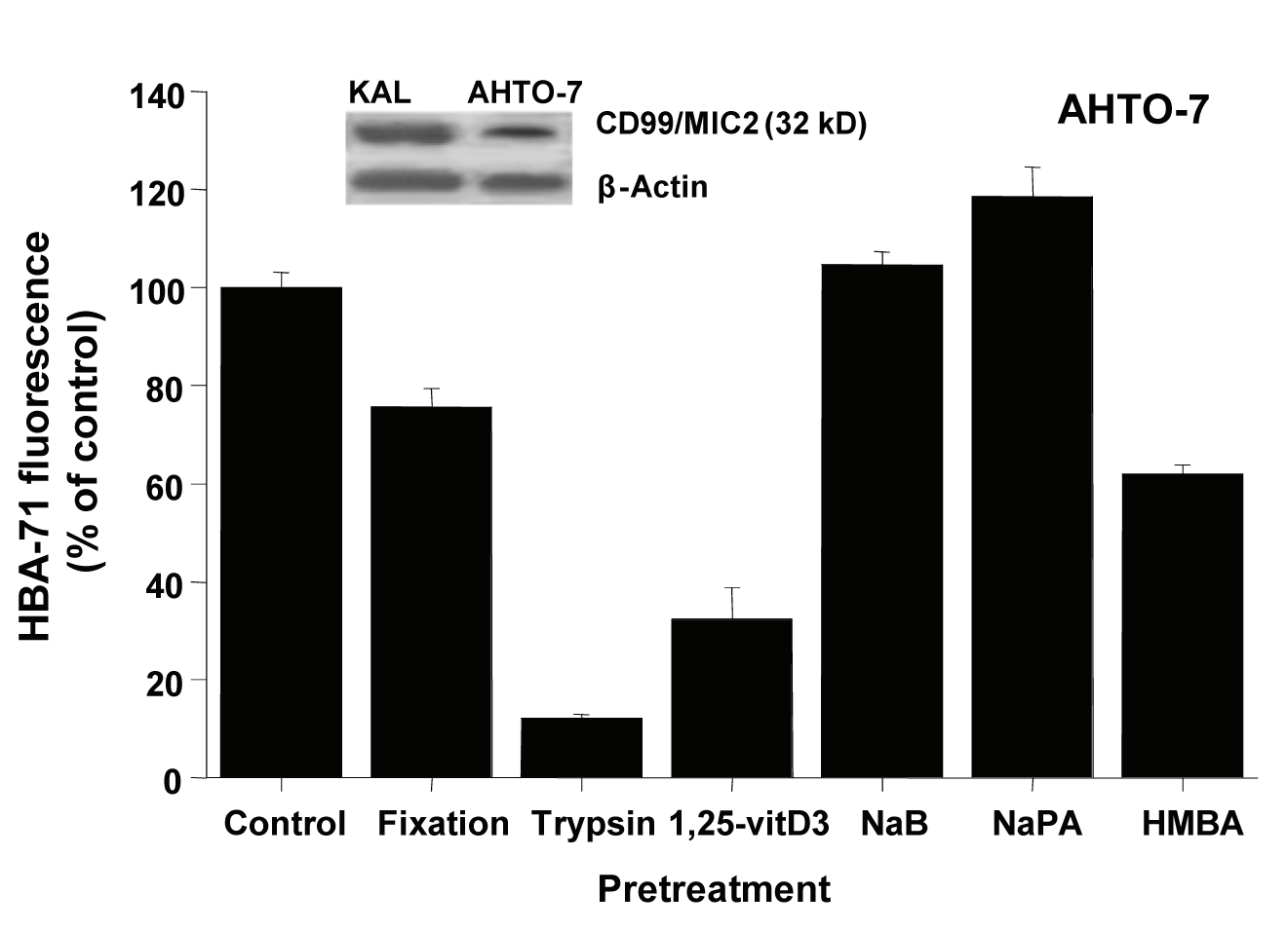
CD99/MIC2 Western blot and effects of fixation, trypsin treatment and differentiation inducers on HBA-71 expression of AHTO-7 osteoblasts. The CD99/MIC2 antigen was blotted with the HBA-71 antibody using extracts of KAL and AHTO-7 cells, respectively. Protein amounts of β-actin are shown as controls. Relative fluorescence intensities are expressed as mean ± SD (n = 3). With the exception of NaB pretreatment, all differences to the control are statistically significant.

Western blots using the HBA-71 antibody revealed protein bands of similar size in protein extracts of KAL and AHTO-7 cells (Fig. 2). Fixation of the osteoblasts with 0.4% paraformaldehyde preserved the CD99/MIC2 antigen to a large extent, whereas mild trypsin treatment resulted in a loss of the cell surface expression of this antigen. It should be noted that the AHTO-7 cells were harvested with the help of Ca^2+^/Mg^2+^-free PBS avoiding enzymatic treatment. Pretreatment of AHTO-7 cells with 1.25-vitD3 and HMBA reduced the cell membrane expression of CD99/MIC2. The differentiation inducer NaB showed no significant effect and NaPA enhanced the antigen expression to a minor extent.

### Effects of differentiation inducers on HBA-71 reactivity of two EFT cell lines

**Figure 3:**
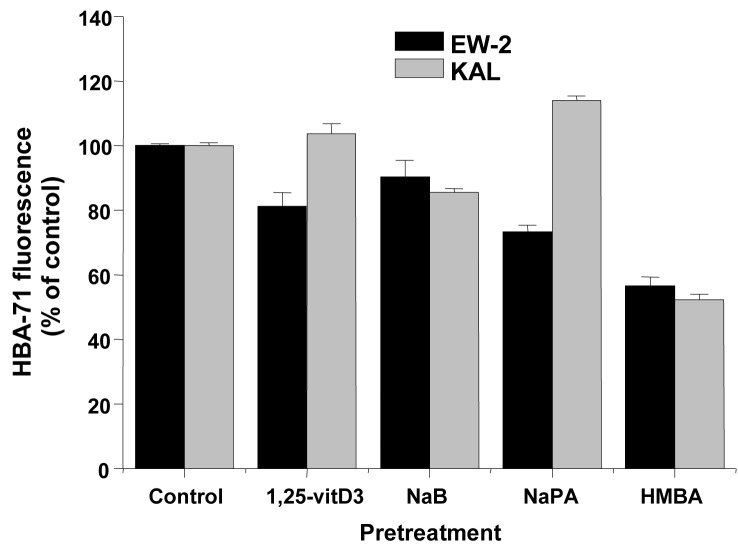
Effects of differentiation inducers on HBA-71 immunofluorescence of EW-2 and KAL EFT cells. Relative fluorescence intensities are expressed as mean ± SD (n = 3). All differences to the respective controls are statistically significant.

The same inducers of differentiation which were applied to AHTO-7 cells were investigated for their effect in regard to CD99/MIC2 expression in the EFT cell lines EW-2 and KAL. All compounds reduced the expression of the HBA-71 CD99/MIC2 antigen of EW-2 cells significantly (Fig. 3). The situation proved to be different in KAL cells, with no activity of 1, 25-vitD3, downregulation of the antigen induced by NaB and HMBA and upregulation by NaPA.

### Effects of differentiation inducers on ALP activity of AHTO-7 and EFT cells

**Figure 4:**
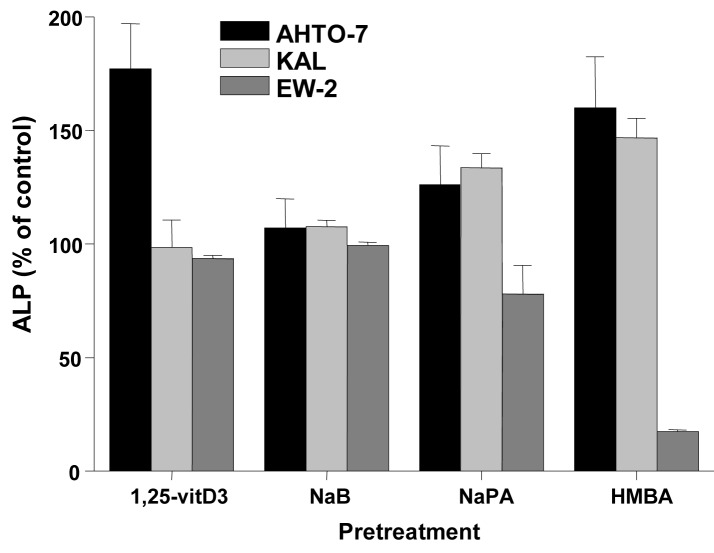
Changes in cellular ALP activity in response to differentiation inducers of AHTO-7, KAL and EW-2 cells. Activity of ALP is expressed as mean ± SD (n = 3). All differences to the control are statistically significant, except for 1, 25-vitD3 and NaB treatment of EW-2 cells.

Changes of ALP activity of the cells in response to inducers were determined as marker of differentiation. Treatment of the AHTO-7 and KAL cells with NaB (except EW-2), NaPA and HMBA resulted in an induction of ALP, whereas in EW-2 cells NaPA and HMBA revealed a decrease in ALP activity. (Fig. 4)

## Discussion

Ewing’s sarcoma (ES) was initially believed to be of perivascular endothelial origin [2]. The Ewing’s sarcoma family of tumors (EFT) includes ES of bone, extraosseous ES, peripheral neuroectodermal tumor of bone (PNET) and malignant small cell tumor of the thoracopulmonary region (Askin’s tumor), all of which are now known to be neoplasms of neuroectodermal origin [8]. The degree of neuronal differentiation is used for subclassification of the EFT as classical ES, which is characterized by minimal signs of neural differentiation, and neural PNET. Classical ES and PNET possessing variable degrees of differentiation are now known to be the same tumor type, defined by a translocation between the EWS gene on chromosome 22 with one of three ETS-like genes, especially the Fli1 gene on chromosome 11 [1]. Nearly all cases of ES contain the (11; 22) (q24; q12) chromosomal translocation that encodes the EWS/Fli1 oncoprotein [9, 10]. Because of the combination of the separate domains the EWS-Fli1 fusion protein acts as an aberrant transcription factor whose expression results in cellular transformation [11].

The cellular origin of ES, a malignancy of bone or soft tissue, is highly debated [12]. CD99 is an integral 32 kD transmembrane glycoprotein encoded by the MIC2 gene with particularly strong expression on T-cell-lineage cells and cells of the EFT. The significance of CD99/MIC2 expression in EFT and its relevance for the histogenetic origin of these tumors has not been characterized so far. Most likely EFTs originate from CD99/MIC2 positive precursor lineage cells and retain expression of the antigen due to its indispensability for maintaining tumor growth. CD99/MIC2 is involved in cell-cell adhesion during hematopoietic cell differentiation, apoptosis of immature thymocytes and transport of transmembrane proteins [13].

Immunohistochemical reactivity of anti-CD99/MIC2 antibodies were reported for bone tissues apparently free of ES; however, this staining was attributed to abnormalities not associated with tumor metastases [14]. Several groups developed immortalized cell lines derived from normal bone tissue to overcome nonclonality and limited supply and proliferation of explant cultures [7, 15]. We tested AHTO-7 cells, an osteoblastic cell line derived from normal bone tissue of a 65 year old male by SV40 large T antigen transformation, for expression of CD99/MIC2. Osteoblasts are thought to originate from mesenchymal stem cells of the colony-forming unit fibroblast lineage that produces fibroblasts, myoblasts, adipocytes and chondrocytes and differentiate to either lining cells or osteocytes terminally [16]. These skeletal cells are responsible for synthesis, deposition and mineralization of the extracellular bone matrix. Stem and primitive osteoprogenitors as well as related mesenchymal precursors arise during embryogenesis and at least some of them appear to persist in the adult organism, where they contribute to the replacement of osteoblasts in bone turnover.

According to our data, AHTO-7 osteoblasts exhibited high HBA-71 reactivity, which was resistant to fixation and sensitive to trypsin, as had been characterized for EFT cells previously [3]. Therefore, expression of CD99/MIC2 is only detectable if trypsin treatment, a routine procedure for maintenance and subcultivation of the adherent osteoblast line, is omitted. Induction of differentiation using 1, 25-vitD3 or the differentiation inducer HMBA resulted in increased ALP activity and was followed by marked downregulation of HBA-71 binding [17]. Consistent with the recognition of CD99/MIC2, Western blot analysis demonstrated reactivity of HBA-71 with a 32 kD protein in human AHTO-7 cells. In agreement with the role of CD99/MIC2 antigen in the maturation of bone marrow stem cells to mature lymphocyte subpopulation via cortical thymocytes, this antigen seems to constitute a differentiation marker in the lineage leading from mesenchymal stem cells to differentiated osteocytes.

EWS-Fli1 is important for the maintenance of tumor growth and thus, antisense modulation of this fusion protein effects decreased growth. The chimeric fusion gene EWS/Fli1 is detected in the majority of ES, the second most common malignant bone tumor of childhood. Although 80% of ES originate in skeletal sites, the remainder can arise at almost any soft tissue location. The lineage of the cell developing the EWS/Fli1 gene fusion has not been fully characterized but is generally considered to be of either mesenchymal or neural crest origin [18]. The phenotypes of different EWS-Fli1-silenced ES cell lines converge toward that of mesenchymal stem cells. Moreover, ES cell lines can differentiate along the osteogenic or adipogenic lineage upon EWS-Fli1 silencing, when incubated in appropriate differentiation media. Interestingly, C2C12 myoblasts transfected with EWS/Fli1 cells constitutively expressed the bone lineage marker ALP and were ALP-positive in histochemistry but did not show any other evidence of bone lineage commitment [18].

Our results show that CD99/MIC2 is a differentiation antigen of human osteoblasts regulated by 1, 25-vitD3 and the potent inducer HMBA. The Runt domain transcription factor Runx2 (AML-3 and Cbfa1) is essential for osteoblast development, differentiation and bone formation [19]. Runx2 positively or negatively regulates osteoblast gene expression and HDAC3 interacts with the N-terminus of Runx2. During osteogenesis total HDAC enzymatic activity is decreased with significant reduction in HDAC1 expression [20]. Suppression of HDAC activity by the inhibitor NaB accelerated osteogenesis by induction of osteoblast marker genes including osteopontin and ALP [21]. Increasing knowledge about mechanisms controlling osteoblast-specific gene expression led to the identification of Cbfa1 as a key regulator of osteoblast differentiation [22]. To identify such Cbfa1-responsive genes the SaOs-2 sarcoma cell line was stably transfected with a dominant-negative mutant of Cbfa1. Four new genes suppressed by Cbfa1, namely CD99/MIC2 and SelM, elF-4A1 and RPS24, linked to a global change in cellular metabolism and growth, were detected. This observation was regarded as further evidence of the relation between the osteoblast lineage and EFT.

Expression of EWS-Fli1, the fusion protein on which ES cells depend, was shown to be negatively influenced by HDAC inhibitors [23, 24]. Results indicated that EWS-Fli1 deregulated histone acetylation through both repression of histone acetyltransferase and enhancement of HDAC activities in EFT cells [23]. A series of preclinical studies demonstrated activity of HDAC inhibitors against ES in vitro and in vivo [25]. A panel of polar HDAC inhibitors were developed with activity increasing in the order NaB < NaPA < HMBA. In particular, HMBA is a hybrid polar compound, which was shown to induce terminal differentiation of transformed cells of several solid tumor and leukemic cell lines [26]. However, dose-limiting toxicity of the drug prevented its clinical administration and led to the development of related hybrid polar compounds such as suberoylanilide hydroxamic acid (SAHA) that induced differentiation of transformed cells more effectively [27].

Modern molecular profiling experiments indicate that Ewing’s tumors originate from mesenchymal precursors in young individuals [28]. EWS-Fli1 alters the morphology of mesenchymal cells and prevents lineage specification. Fli sequences within EWS-Fli fusion protein are responsible for interactions with Runx2. EWS-Fli blocks the ability of Runx2 to induce osteoblast differentiation of a mesenchymal progenitor cell and seems to prevent expression of CD99/MIC2. Disrupting interactions between Runx2 and EWS-Fli1 may promote differentiation of the tumor cells. Thus, current evidence indicates that the fusion gene EWS-Fli1 may induce the transformation of mesenchymal stem cells into ES cells [12, 29]. Tirode and colleagues showed that the profile of ES lines converge to that of normal mesenchymal stem cells after EWS-Fli1 abrogation. Moreover, silenced lines could recover part of their differentiation potential primarily toward adipocytes and osteoblasts. Other data indicated that ES cells express neural markers, compatible with the transformation of neuroectodermally derived mesenchymal stem cells or might result from EWS-Fli1-driven neuroectodermal differentiation in transformed mesenchymal stem cells [30]. Therefore, the presented results again correlate ES with osteoblasts and osteoblastic differentiation by analysis of the expression of the CD99/MIC2 antigen expression.

### Conclusion

CD99/MIC2, as detected by the HBA-71 monoclonal antibody, is expressed as differentiation antigen in the human osteoblast cell line AHTO-7. Induction of osteoblastic differentiation in AHTO-7 by 1, 25-vitD3 or HMBA resulted in downregulation of CD99/MIC2, similar to the effect of the HDAC inhibitor HMBA in KAL PNET cells. In good accordance with other evidence linking the origin of EFT with mesenchymal stem cells, our results are in line with the development of ES from precursor cells of the osteoblastic lineage.
